# The prognostic significance of KLRB1 and its further association with immune cells in breast cancer

**DOI:** 10.7717/peerj.15654

**Published:** 2023-07-24

**Authors:** Ning Xu, Xiangyu Meng, Hongyu Chu, Zhaoying Yang, Yan Jiao, Youjun Li

**Affiliations:** 1 Department of Human Anatomy, Jilin University, Changchun, Jilin, China; 2 Department of Breast Surgery, China-Japan Union Hospital of Jilin University, Changchun, Jilin, China; 3 Department of Gastrointestinal, Colorectal and Anal Surgery, China-Japan Union Hospital of Jilin University, Changchun, Jilin, China; 4 Department of Hepatobiliary and Pancreatic Surgery, General Surgery Center, The First Hospital of Jilin University, Changchun, Jilin, China

**Keywords:** Killer cell lectin-like receptor B1 (KLRB1), Breast cancer, Prognosis, Biomarker, Tumor immune microenvironment, Macrophages

## Abstract

**Background:**

Killer cell lectin-like receptor B1 (KLRB1) is an important member of the natural killer cell gene family. This study explored the potential value of KLRB1 as a breast cancer (BC) biomarker and its close association with the tumor immune microenvironment during the development of BC.

**Methods:**

We examined the differential expression of KLRB1 in pan-cancer. Clinical and RNA-Seq data from BC samples were evaluated in The Cancer Genome Atlas (TCGA) and validated in Gene Expression Omnibus (GEO) datasets and by immunohistochemistry (IHC) staining. The relationship between KLRB1 and clinical parameters was explored through Chi-square tests. The diagnostic value of KLRB1 was evaluated using a receiver operating characteristic (ROC) curve. Survival analysis was tested by Kaplan-Meier curves to demonstrate the relationship between KLRB1 and survival. Univariable and multivariate cox regression analyses were carried out as well. The analysis of immune infiltration level and gene set enrichment analysis (GSEA) were conducted to examine KLRB1’s mechanism during the progression of BC. We used the Tumor Immune Estimation Resource (TIMER), the Cancer Single-cell Expression Map (CancerSCEM) database, the Tumor Immune Single-cell Hub (TISCH) database, and the Cell-type Identification by Estimating Relative Subsets of RNA Transcripts (CIBERSORT) method to explore KLRB1’s association with immune infiltration level and different quantitative distribution of immune cells. The relevant signaling pathways in BC associated with KLRB1 were identified using GSEA.

**Results:**

The expression of KLRB1 was downregulated across the majority of cancers including BC. The lower KLRB1 expression group exhibited shorter relapse free survival (RFS) and overall survival (OS). IHC staining showed that KLRB1 staining was weaker in breast tumor tissues than in paratumors. Additionally, GSEA identified several pathway items distinctly enriched in BC. KLRB1 expression level was also positively related to the infiltrating number of immune cells in BC. Moreover, the CancerSCEM and TISCH databases as well as the CIBERSORT method demonstrated the close relationship between KLRB1 and immune cells, particularly macrophages.

**Conclusion:**

Low KLRB1 expression was considered an independent prognostic biomarker and played an important role in the tumor immune microenvironment of BC patients.

## Introduction

There has been much debate about the relationship between different types of cancers. Breast cancer (BC) is associated with a high mortality rate in women (Donepudi et al., 2014; Hartmann & Loprinzi, 1990). The prognosis of BC is linked to several biomarkers, including estrogen receptors (ERs), progesterone receptors (PRs), human epidermal growth factor receptor-2 (HER2), and Ki-67. However, as precision medicine and personalized treatment methods advance, these markers cannot fulfill all medical requirements (Watkins, 2019). For instance, some patients show resistance to endocrine therapy because of ligand-independent ER reactivation, despite having an ER (+) status (Hanker, Sudhan & Arteaga, 2020). Therefore, new biomarkers are urgently required to improve BC diagnosis and prognosis, as well as for choosing optimal therapeutic strategies.

Killer cell lectin receptor B1 (*KLRB1*) is identical to natural killer cell surface protein P1 (NKRP1), C-type lectin domain family 5-member B (CLEC5B), and CD161. It is a homodimeric C-type-lectin that signifies the link between human and rodent *NKRP1* (Yokoyama & Seaman, 1993). *KLRB1* is mainly expressed in the bone marrow, spleen, and other tissues, with higher levels in mammary lymph nodes and tumors (Fahmi et al., 2010).

Prior studies have reported *KLRB1* as a potential prognostic and immunological marker across tumors using pan-cancer analysis (Cheng et al., 2022). Based on the estimate algorithm, it was determined that *KLRB1* was associated with changes in the tumor microenvironment (TME) and a better prognosis marker of hepatocellular carcinoma (Pan et al., 2020). High *KLRB1* expression could suppress the tumor formation in the gastric cancer mouse model (Adithan et al., 2020). Moreover, some other studies noted the importance of *KLRB1* in several cancers, including esophageal squamous cell carcinoma, cutaneous melanoma, nasopharyngeal carcinoma, T-cell prolymphocytic leukemia, and lung cancer (Domvri et al., 2020; Gilles et al., 2019; Liu et al., 2020; Martinović et al., 2019; Zhang et al., 2020). *KLRB1*’s relationship with BC, including its potential use in immunotherapy, has been explored and verified by qRT-PCR (Weng et al., 2022); however, more in-depth studies are needed to confirm these observations. In this article, we further validated the results using immunochemistry (IHC) staining, analysis of diagnostic capacity, and single cell analysis, and hypothesized that *KLRB1* may be regulated by crucial genes and potential pathways.

In this study, we evaluated the role of *KLRB1* as a potential independent prognostic biomarker by studying its association with the clinicopathologic features of BC and clarified the influence of *KLRB1* on patient survival using public datasets from The Cancer Genome Atlas (TCGA) database. The results were validated by IHC staining using the paired tissue samples. Next, we explored the role of *KLRB1* in the TME as well as the potential of *KLRB1* as a new target of immunotherapy. Finally, we analyzed the specific mechanism by which *KLRB1* influences the occurrence and development of BC by focusing on immune cell infiltration, relative pathways, and related crucial genes.

## Material and Methods

### Pan-cancer analysis of *KLRB1*

In order to widely and swiftly learn the functions of *KLRB1*, we used the Tumor Immune Estimation Resource (TIMER) (https://cistrome.shinyapps.io/timer/) to show the differentiation and expression between normal and cancer tissues. Subsequent analysis of relative survival was conducted through the human protein atlas (https://www.proteinatlas.org/). The inclusion criteria were: *p* < 0.05 and the two curves in the survival analysis could not intercross.

### Data mining and analysis using TCGA database

Clinical information and level three RNA-seq expression data (1,104 tumors and 114 normal samples) were collected from the public TCGA website. Furthermore, R software (version 3.5.3; R Core Team, 2019) was used for data processing. RNA-seq by expectation maximization (RSEM) was applied to analyze RNA-Seq data. The GSE42568 (Clarke et al., 2013) dataset was acquired from the GEO website.

### IHC staining

A total of 23 paired tumors were collected together with the adjacent tissues of BC patients. Following the surgery, patients received a histological diagnosis complying with the criteria of the World Health Organization (WHO) from two separate pathologists. Written informed consent was obtained from all individual participants included in the study and all tissues involved in our study were approved by the China-Japan Union Hospital ethics committee of Jilin University (approval number: 2019022606). As previously described in Yan et al.’s article (2021), IHC staining was adopted to exhibit *KLRB1* expression level across the tissues of BC patients. Specifically, the sections (3 µm-thick) were deparaffinized, followed by rehydration and submergence into ethylenediaminetetraacetic acid (EDTA) for the retrieval of antigen. After the sections were hydrogenated and heated, they were incubated with the media containing bovine serum albumin, followed by incubation with the *KLRB1* antibody (Abcam, Cambridge, MA, USA) overnight (4 °C). The serum from healthy rabbits were taken as the negative control. The sections were washed, then cultivated with the secondary antibody, followed by incubation with horseradish peroxidase-streptavidin complex (Invitrogen, Waltham, MA, USA). After being immersed in 3-amino-9-ethyl carbazole and counterstained with Mayer’s hematoxylin, sections were dehydrated and mounted. Cytoplasm staining was regarded as positive.

### Immune infiltrate analysis based on TCGA database with TIMER

Using the data obtained from TCGA, TIMER was applied for the analysis of the immune infiltrate in order to investigate the expression of *KLRB1* and its relationship with immune infiltrates such as dendritic cells, neutrophils, CD4+ cells, CD8+ cells, B cells, and macrophages in various molecular groups of BC through gene segments.

### Gene set enrichment analysis (GSEA)

A bioinformatics-based protocol, GSEA, was used to determine the statistical significance among the predefined groups of genes and the presence of agreed variations in two different biological states. GSEA classified the genes that were linked to *KLRB1* expression by analyzing the difference of high *KLRB1* expression groups from the low ones. Additionally, the enriched pathways among different phenotypes were categorized by nominal *p*-value and normalized enrichment score (NES). The enrichment terms, including a false discovery rate (FDR) less than 0.25 together with a NOM *p* value less than 0.05, were adopted for the identification of enriched gene sets.

### Single cell analysis using the Cancer Single-cell Expression Map (CancerSCEM) and Tumor Immune Single-cell Hub (TISCH) databases

Based on the GEO databases (GSE148673 and GSE143423) (Gao et al., 2021; Wang et al., 2019), the CancerSCEM database (https://ngdc.cncb.ac.cn/cancerscem/index) provided the results of the distribution of various immune cells in triple negative BC. Furthermore, the GSE114727 (Azizi et al., 2018) and TISCH databases (http://tisch.comp-genomics.org/home/) were used to demonstrate a differently quantitative distribution of immune and stromal cells of *KLRB1* in BC.

### Statistical analysis

*KLRB1* expression among BC patients obtained from the TCGA database was evaluated using the ggplot2 package in R. Boxplots were constructed to determine the differences in discrete variables using Wilcoxon and Kruskal-Wallis tests. The correlations between clinicopathological features and *KLRB1* expression were examined by Chi-square and Fisher’s exact tests. The ROC curve was constructed using the pROC package for assessing the diagnostic capability of *KLRB1*. The best operating system cut-off value (4.858) was obtained from the ROC curve to categorize BC patients into groups with low/high *KLRB1* expression. Kaplan–Meier analysis was performed to compare the rate of relapse-free survival (RFS) as well as the rate of overall survival (OS) between the high and low *KLRB1* expression groups through the survival package. The independent influencing factors OS and RFS were analyzed using univariate and multivariate Cox analysis. Additionally, the correlation of *KLRB1* with other genes in the RNA-seq data was evaluated using the “cor.test” package with Spearman rank correlation analysis. By using the CIBERSORT method (Newman et al., 2015) in R, a computational method to determine leukocyte expression among bulk tumor transcriptomes, we evaluated the complex relationship of 22 distinct subsets of leukocytes with the expression of *KLRB1* using the “pheatmap” and “vioplot” packages. The correlation between two distinct leukocyte subsets was assessed using the “corrplot” package. We also showed the relationship of 22 leukocyte subsets with BC survival using the “survival” package. *P* values less than 0.05 were considered the threshold for the significance.

## Results

### Pan-cancer analysis of *KLRB1* expression

**Figure 1:**
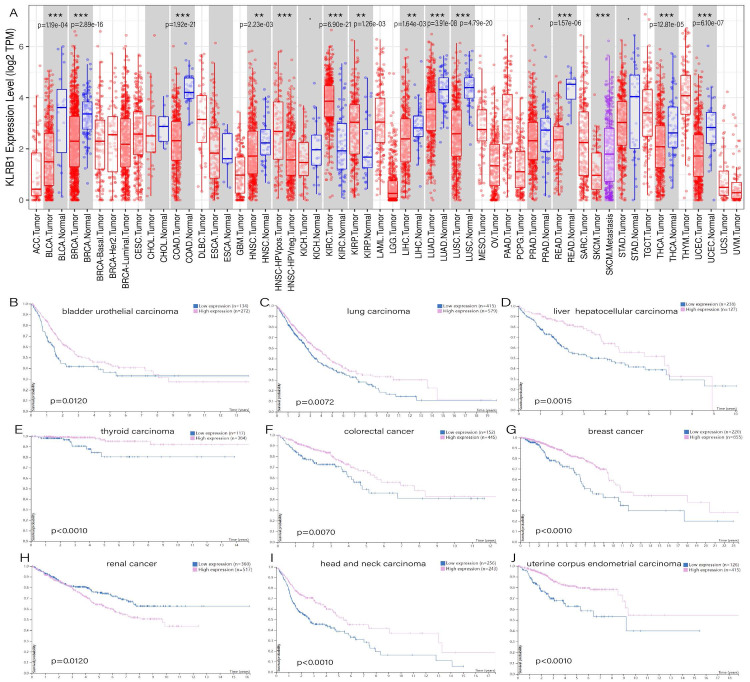
(A) Expression difference between normal and tumor tissues, meaningful cancer types including bladder urothelial carcinoma, breast invasive carcinoma, colon adenocarcinoma, rectum adenocarcinoma, head and neck squamous cell carcinoma, liver hepatocellular carcinoma, lung adenocarcinoma, lung squamous cell carcinoma, thyroid carcinoma, uterine corpus endometrial carcinoma, kidney renal clear cell carcinoma, and kidney renal papillary cell carcinoma. *P*-value significant codes: 0 ≤ ∗∗∗ < 0.001 ≤ ∗∗ < 0.01 ≤ ∗ < 0.05 ≤ . < 0.1. The survival analysis of cancers of bladder urothelial carcinoma (B), lung carcinoma (C), liver hepatocellular carcinoma (D), thyroid carcinoma (E), colorectal cancer (F), breast cancer (J), renal cancer (H), head and neck carcinoma (I) and uterine corpus endometrial carcinoma (J).

TIMER showed distinct differences existing between normal tissues and tumor tissues, especially in bladder urothelial carcinoma (*p* = 1.19e−04), breast invasive carcinoma (*p* = 2.89e−16), colon adenocarcinoma (*p* = 1.92e−21), rectum adenocarcinoma (*p* = 1.57e−06), head and neck squamous cell carcinoma (*p* = 2.23e−03), liver hepatocellular carcinoma (*p* = 1.64e−03), lung adenocarcinoma (*p* = 3.91e−08), lung squamous cell carcinoma (*p* = 4.79e−20), thyroid carcinoma (*p* = 2.81e−05), uterine corpus endometrial carcinoma (*p* = 6.10e−07), and two types of renal carcinoma: kidney renal clear cell carcinoma (*p* = 6.90e−21) and kidney renal papillary cell carcinoma (*p* = 1.26e−03) (Fig. 1A). The survival analysis showed that all of the cancers mentioned above possessed meaningful outcomes. Higher *KLRB1* expression was linked to a lower survival rate only in renal carcinoma, while all of the others showed a better prognosis (Figs. 1B–1J).

### Patient characteristics

The clinicopathological features and *KLRB1* gene expression information for the 1,104 breast tumor samples from TCGA are presented in Table S1, and include sample type, histological type, vital status, molecular subtype, status of ER, PR, HER-2, TNM stage, lymph node status, and clinical stage.

### Relationship between clinical features of BC patients and *KLRB1* expression

**Figure 2:**
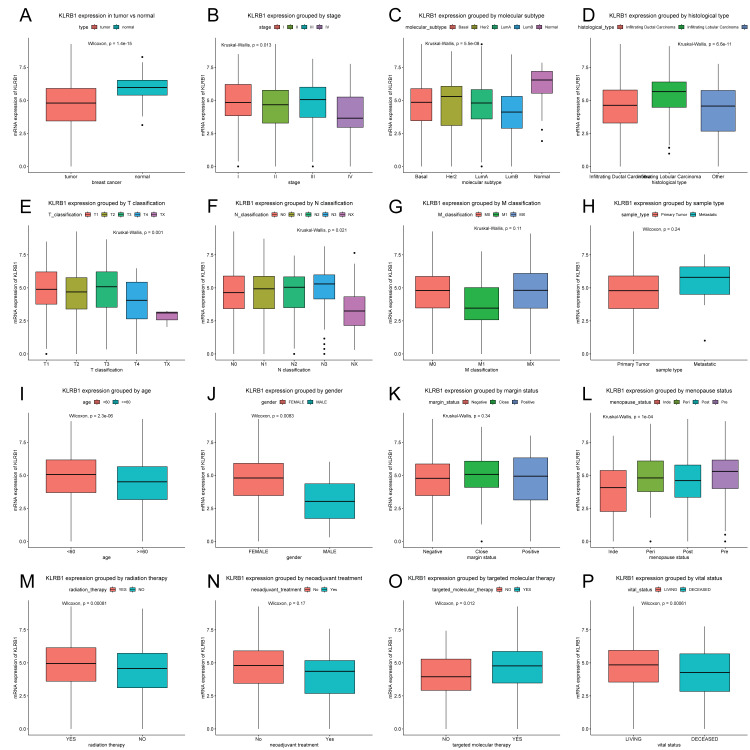
KLRB1 expression in breast tumors. The expression of KLRB1 is lower in tumors (A). Differences in KLRB1 expression were shown in clinical stage (*p* = 0.0130) (B), molecular subtype (*p* < 0.0001) (C), histological type (*p* < 0.0001) (D), T classification (*p* = 0.0010) (E), N classification (*p* = 0.0210) (F), M classification (G), sample type (H), age (*p* < 0.0001) (I), gender (*p* = 0.0083) (J), margin status (K), menopause status (*p* < 0.0001) (L), radiation therapy (*p* = 0.0008) (M), neoadjuvant treatment (N), targeted molecular therapy (*p* = 0.0120) (O) and vital status (*p* = 0.0095) (P).

Patients were divided into two groups (high/low *KLRB1* expression) using the optimal cutoff value of 4.858 based on the ROC curve. In contrast to normal breast tissues (*n* = 114), *KLRB1* expression in tumors (*n* = 1,104) was found to be significantly lower by Wilcoxon and Kruskal-Wallis tests (*p* < 0.0001). *KLRB1* expression differed depending on stage, molecular subtype, histological type, T classification, N classification, age, gender, menopause status, radiation therapy, targeted molecular therapy, and vital status (all *p* < 0.05) (Fig. 2).

Next, the results of Chi-square/Fisher’s exact test showed the correlation of *KLRB1* expression with clinical characteristics of BC. Table 1 shows significant correlations between *KLRB1* expression and the following characteristics: age, histological type, molecular subtype, ER status, menopause status, T classification, N classification, stage, radiation therapy, vital status, OS, RFS, and targeted molecular therapy (all *p* < 0.05). Specifically, in T classification, M classification, and clinical stage, T4, M1, and stage IV had the lowest *KLRB1* expression among the other T classifications and stages (Table 1).

**Table 1:** Relationships between KLRB1 expression and clinical characteristics in breast cancers using chi-square and Fisher’s exact tests.

Parameter	Variable	Number	High	Hprop	Low	Lprop	X^2^	*P* value	Fisher
Age	<60	589	407	(69.10)	182	(30.90)	7.5489	**0.0055**	0.0064
	> =60	513	314	(61.21)	199	(38.79)			
Gender	Female	1090	716	(65.69)	374	(34.31)	3.0278	0.1269	0.1229
	Male	12	5	(41.67)	7	(58.33)			
Histological type	Infiltrating Ductal Carcinoma	790	489	(61.90)	301	(38.10)	31.6993	**0.0005**	0.0005
	Infiltrating Lobular Carcinoma	204	168	(82.35)	36	(17.65)			
	Other	107	64	(59.81)	43	(40.19)			
Molecular subtype	Basal	142	96	(67.61)	46	(32.39)	22.9660	**0.0005**	0.0010
	HER2	67	46	(68.66)	21	(31.34)			
	LumA	422	285	(67.54)	137	(32.46)			
	LumB	194	98	(50.52)	96	(49.48)			
	Normal	24	20	(83.33)	4	(16.67)			
ER	Indeterminate	2	2	(100.00)	0	(00.00)	5.3216	0.0565	**0.0495**
	Negative	239	171	(71.55)	68	(28.45)			
	Positive	813	523	(64.33)	290	(35.67)			
PR	Indeterminate	4	4	(100.00)	0	(00.00)	2.2839	0.3543	0.4108
	Negative	345	231	(66.96)	114	(33.04)			
	Positive	704	461	(65.48)	243	(34.52)			
HER2	Equivocal	180	112	(62.22)	68	(37.78)	4.9448	0.1709	0.1784
	Indeterminate	12	9	(75.00)	3	(25.00)			
	Negative	565	392	(69.38)	173	(30.62)			
	Positive	164	103	(62.80)	61	(37.20)			
Menopause status	Inde	34	17	(50.00)	17	(50.00)	12.9076	**0.0045**	0.0050
	Peri	40	28	(70.00)	12	(30.00)			
	Post	706	447	(63.31)	259	(36.69)			
	Pre	231	171	(74.03)	60	(25.97)			
T classification	T1	281	194	(69.04)	87	(30.96)	12.9644	0.0130	**0.0140**
	T2	640	411	(64.22)	229	(35.78)			
	T3	138	96	(69.57)	42	(30.43)			
	T4	40	20	(50.00)	20	(50.00)			
	TX	3	0	(00.00)	3	(100.0)			
N classification	N0	516	334	(64.73)	182	(35.27)	12.4067	**0.0120**	0.0200
	N1	367	239	(65.12)	128	(34.88)			
	N2	120	81	(67.50)	39	(32.50)			
	N3	79	60	(75.95)	19	(24.05)			
	NX	20	7	(35.00)	13	(65.00)			
M classification	M0	917	608	(66.30)	309	(33.70)	6.3475	**0.0440**	0.0540
	M1	22	9	(40.91)	13	(59.09)			
	MX	163	104	(63.80)	59	(36.20)			
Stage	I	182	128	(70.33)	54	(29.67)	15.1199	**0.0065**	0.0050
	II	626	395	(63.10)	231	(36.90)			
	III	252	177	(70.24)	75	(29.76)			
	IV	20	9	(45.00)	11	(55.00)			
	X	14	5	(35.71)	9	(64.29)			
Lymph node status	No	28	17	(60.71)	11	(39.29)	0.5509	0.5467	0.5384
	Yes	697	470	(67.43)	227	(32.57)			
Margin status	Close	31	23	(74.19)	8	(25.81)	1.0288	0.5947	0.6402
	Negative	922	605	(65.62)	317	(34.38)			
	Positive	79	53	(67.09)	26	(32.91)			
Vital status	Deceased	155	80	(51.61)	75	(48.39)	15.2153	**0.0005**	0.0002
	Living	947	641	(67.69)	306	(32.31)			
Radiation therapy	No	445	274	(61.57)	171	(38.43)	4.3067	**0.0395**	0.0390
	Yes	557	378	(67.86)	179	(32.14)			
Neoadjuvant treatment	No	1088	713	(65.53)	375	(34.47)	0.7754	0.3878	0.3901
	Yes	13	7	(53.85)	6	(46.15)			
Targeted molecular therapy	No	46	20	(43.48)	26	(56.52)	8.6985	**0.0040**	0.0040
	Yes	533	348	(65.29)	185	(34.71)			
Sample type	Metastatic	7	5	(71.43)	2	(28.57)	0.1100	1.0000	1.0000
	Primary Tumor	1097	718	(65.45)	379	(34.55)			
OS	0	933	632	(67.74)	301	(32.26)	14.5851	**0.0010**	0.0002
	1	154	80	(51.95)	74	(48.05)			
RFS	0	816	551	(67.52)	265	(32.48)	5.8253	**0.0180**	0.0220
	1	96	53	(55.21)	43	(44.79)			

**Notes.**

Abbreviations: Bold values of *P* < 0.05 indicate statistically significant correlations. When the expected frequency is lower than 1, the Fisher’s exact test result is selected.

### Diagnostic capability of *KLRB1*

**Figure 3:**
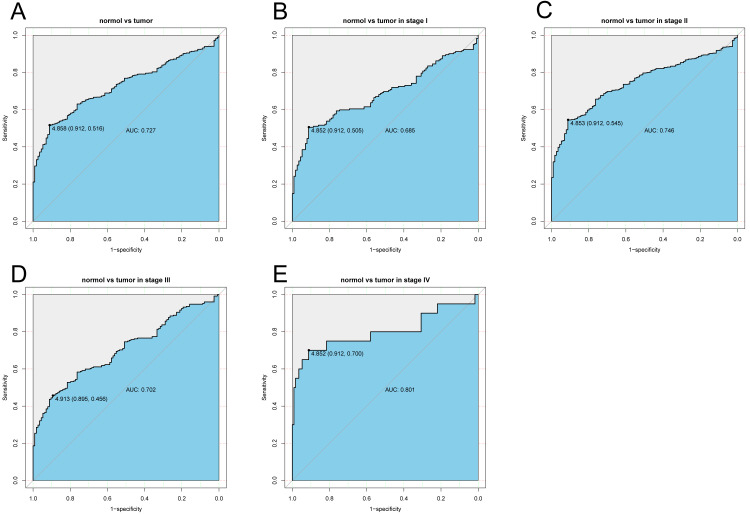
The ROC curve of KLRB1 in breast cancer. ROC curve for KLRB1 expression in breast cancer and normal tissues (A) (AUG: 0.727). Subgroup analyses: Stage I (B) (AUG: 0.685), Stage II (C) (AUG: 0.746), Stage III (D) (AUG: 0.702), and Stage IV (E) (AUG: 0.801). Different stages of breast cancer also showed certain diagnostic value. Abbreviations: AUC, area under the curve; ROC, receiver operating characteristic.

The diagnostic capability of *KLRB1* was evaluated by analyzing the ROC curve. The AUC was 0.727, indicating that *KLRB1* demonstrated a good diagnostic capability. Similar diagnostic capability was also observed in different cancer stages (AUCs for stage I, II, III, and IV were 0.6850, 0.7460, 0.7020, and 0.8010, respectively) (Fig. 3). Stage IV had the highest diagnostic capacity (AUC: 0.801). This result showed a correlation between *KLRB1* expression and stages (Fig. 2), with the lowest *KLRB1* expression level detected in stage IV.

### Association of poor OS with low expression of *KLRB1*

**Figure 4:**
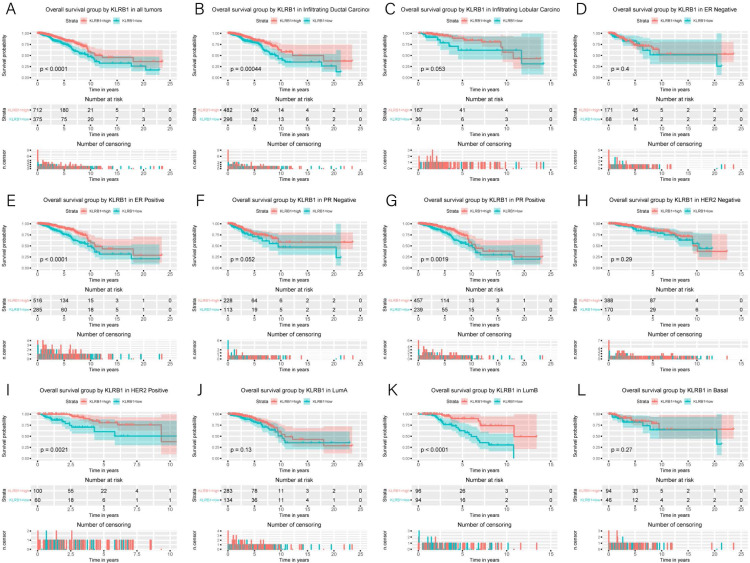
Kaplan-Meier curves of OS in breast cancer according to KLRB1 expression in breast cancer tissues. Overall survival analysis (A) and subgroup analyses of histological type (B and C), ER (D and E), PR (F and G), HER-2 (H and I), and molecular subtype (J, K, and L). Low KLRB1 expression showed a relationship with poor overall survival (*p* < 0.0001).

Kaplan–Meier curves were constructed to evaluate the relationship between *KLRB1* expression and OS. As shown by the log-rank test results (Fig. 4), low *KLRB1* expression was closely related to poor OS. The following were also shown to correlate with low *KLRB1* expression: poor OS among all tumors, ER (+) status, PR (+) status, HER-2 (+) status, as well as luminal B subtype (all *p* < 0.05).

**Figure 5:**
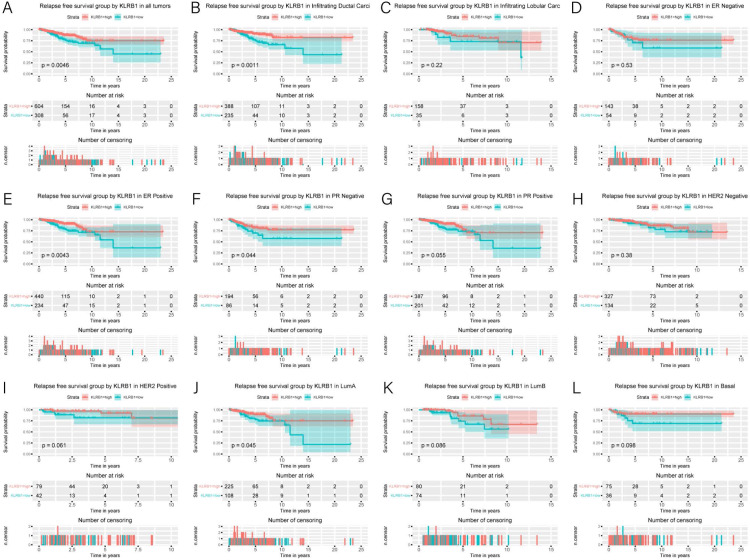
Kaplan-Meier curves of RFS in breast cancer according to KLRB1 expression in breast cancer tissues. Relapse free survival analysis (A) and subgroup analyses of histological type (B and C), ER (D and E), PR (F and G), HER-2 (F and G), and molecular subtype (J, K, and L). Low KLRB1 expression showed a relationship with poor relapse free survival (*p* = 0.0046).

The following characteristics’ association with *KLRB1* expression was summarized using a model based on Cox proportional hazards regression (Table 2). Univariate and multivariate Cox regression analyses showed *KLRB1* (HR: 2.37, 95% CI [1.49–3.76], *p* < 0.0010) to be an independent factor influencing OS. Age (HR: 2.12, 95% CI [1.32–3.39], *p* = 0.0020) together with stage (HR: 1.97, 95% CI [1.52–2.55], *p* < 0.0010) were also found to be independent factors (Fig. 5A).

**Table 2:** Univariate and multivariate analyses of the correlations of KLRB1 expression with OS in breast cancer patients.

Parameters	HR	CI 95%	*P* value	HR	CI 95%	*P* value
Age	1.91	1.39–2.63	0.000	2.12	1.32–3.39	**0.0020**
Histological type	0.93	0.74–1.17	0.543			
Molecular subtype	1.01	0.88–1.16	0.901			
ER	0.85	0.71–1.02	0.074			
PR	0.87	0.73–1.03	0.096			
HER2	1.29	1.05–1.57	0.013	1.22	0.98–1.52	0.0720
Menopause status	1.16	0.94–1.43	0.165			
Stage	1.64	1.4–1.91	0.000	1.97	1.52–2.55	**0**
Lymph node status	1.10	0.93–1.3	0.274			
Margin status	1.42	1.11–1.81	0.005	1.05	0.76–1.47	0.7570
KLRB1	1.88	1.37–2.59	0.000	2.37	1.49–3.76	**0**

**Notes.**

Abbreviations HR Hazard Ratio CI confidence interval

Bold values of *P* < 0.05 indicate statistically significant correlations.

### Association of poor RFS with low expression of *KLRB1*

**Figure 6:**
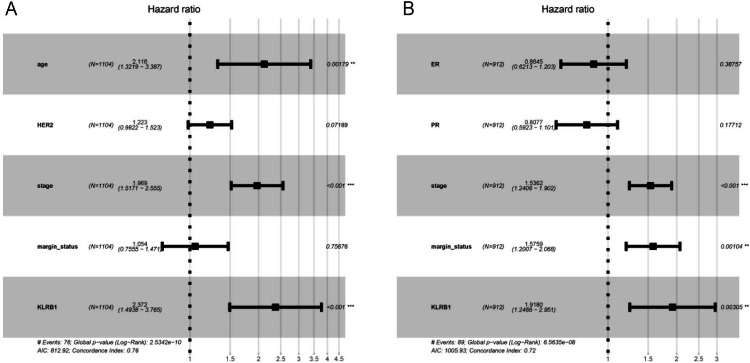
Multivariate Cox analysis of KLRB1 expression and other clinical pathological factors in OS (A) and RFS (B).

Low *KLRB1* expression correlated with poor RFS (Fig. 6) in all tumors, infiltrating duct carcinoma, ER (+) cancers, ER (-) cancers, as well as luminal A subtype (all *p* < 0.05).

Cox analysis indicated that reduced *KLRB1* expression functioned as an independent indicator of poor RFS (HR: 1.92, 95% CI [1.25–2.95], *p* = 0.0030) (Table 3). Other indicators had a marginal status (HR: 1.58, 95% CI [1.20–2.07], *p* = 0.0010) and stage (HR: 1.54, 95% CI [1.24–1.90], *p* < 0.0010) (Fig. 5B).

**Table 3:** Univariate and multivariate analyses of the correlations of KLRB1 expression with RFS in breast cancer patients.

Parameters	HR	CI 95%	*P* value	HR	CI 95%	*P* value
Age	1.45	0.97–2.16	0.072			
Histological type	0.86	0.65–1.14	0.29			
Molecular subtype	0.99	0.82–1.2	0.945			
ER	0.78	0.63–0.97	0.026	0.86	0.62–1.2	0.3880
PR	0.78	0.64–0.96	0.019	0.81	0.59–1.1	0.1770
HER2	0.93	0.7–1.22	0.596			
Menopause status	0.95	0.74–1.22	0.713			
Stage	1.71	1.4–2.08	0.000	1.54	1.24–1.9	**0**
Lymph node status	0.86	0.7–1.06	0.159			
Margin status	1.59	1.23–2.06	0.000	1.58	1.2–2.07	**0.0010**
KLRB1	1.78	1.19–2.66	0.005	1.92	1.25–2.95	**0.0030**

**Notes.**

Abbreviations HR Hazard Ratio CI confidence interval

Bold values of *P* < 0.05 indicate statistically significant correlations.

### Characteristics of BC patients and *KLRB1* expression: GEO database analysis

**Figure 7:**
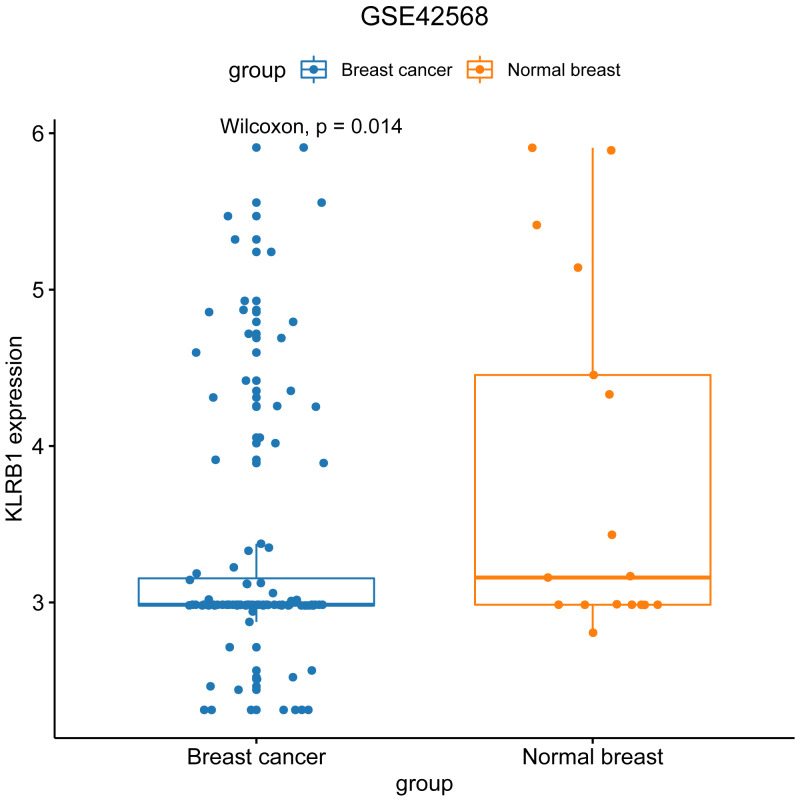
The relevance of KLRB1 expression in the GEO database. KLRB1 expression in normal breast tissues was higher than in breast cancer tissues (*p* = 0.0140).

We used the GEO database to examine various patient classifications based on their pathological *KLRB1* expression and clinical traits. The result was similar to that of our previous research (Fig. 7). *KLRB1* expression was significantly lower in BC samples (*p* = 0.0140) than in healthy samples.

### Distribution of immune cells based on single cell analysis with CancerSCEM and TISCH

**Figure 8:**
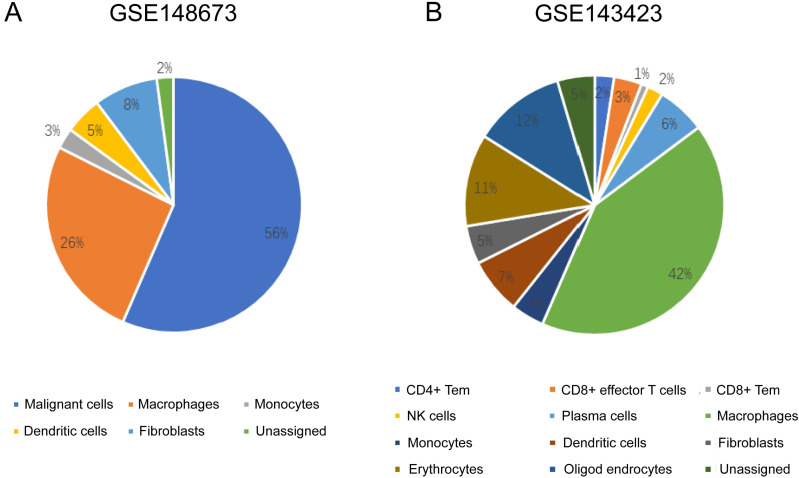
The quantitative distribution of immune cells in breast cancer datasets GSE128673 (A) and GSE143423 (B). The results are based on the Cancer Single-cell Expression Map database (https://ngdc.cncb.ac.cn/cancerscem/index).

**Figure 9:**
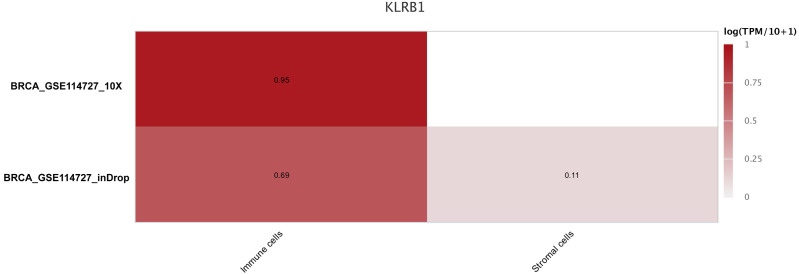
The different expression of KLRB1 between immune cells and stromal cells. KLRB1 possesses higher expression in immune cells than in stromal cells. The results are based on the TISCH database (http://tisch.comp-genomics.org/home/).

The sequencing results from the CancerSCEM database revealed that the majority of immune cells consisted of macrophages (56% and 42% in GSE148673 and GSE143423, respectively, in triple-negative BC; Fig. 8). Moreover, analysis of TISCH database revealed that in contrast to stromal cells, *KLRB1* expression in immune cells was much higher, demonstrating its close relevance with the TME (Fig. 9).

### Relationship between tumor-infiltrating immune cells and *KLRB1* expression: Analysis using TIMER

**Figure 10:**
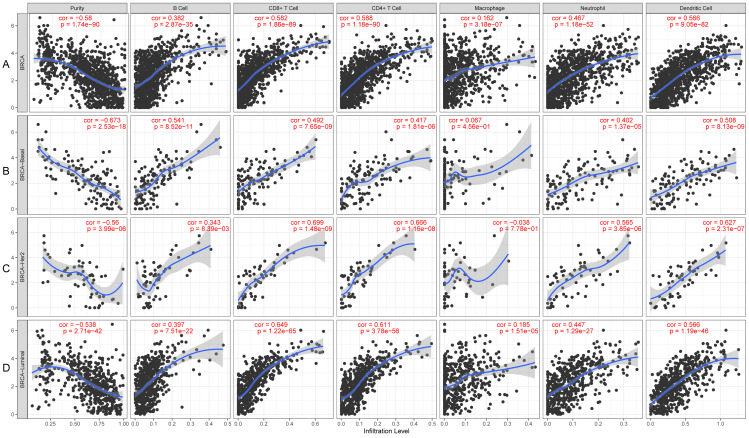
The expression levels of KLRB1 are negatively correlated with tumor purity (A) in basal-like (B), HER2-enriched (C), and luminal (D) breast cancer. The KLRB1 expression level had significant positive correlations with infiltrating levels of B cells (*p* = 2.11e − 30, *r* = 0.355), CD8 + T cells (*p* = 9.45e − 62, *r* = 0.494), CD4 + T cells (*p* = 9.88e − 70, *r* = 0.527), macrophages (*p* = 1.56e − 03, *r* = 0.101), neutrophils (*p* = 2.71e − 36, *r* = 0.392), and dendritic Cells (*p* = 4.46e − 63, *r* = 0.506) in breast cancer.

*KLRB1* expression levels correlated with tumor purity in BC (*p* = 2.66e−88, *r* =  − 0.574), as well as with luminal type (*p* = 2.35e−42, *r* =  − 0.538), HER2-enriched (*p* = 2.22e−05, *r* =  − 0.522), and basal-like (*p* = 2.39e−17, *r* =  − 0.658) BC. Moreover, a positive association of *KLRB1* expression level with the levels of infiltrating B cells (*p* = 2.11e−30, *r* = 0.355), CD8+ cells (*p* = 9.45e −62, *r* = 0.494), CD4+ cells (*p* = 9.88e−70, *r* = 0.527), macrophages (*p* = 1.56e−03, *r* = 0.101), neutrophils (*p* = 2.71e−36, *r* = 0.392), and dendritic cells (*p* = 4.46e−63, *r* = 0.506) was observed. A similar trend was noted for different molecular types of BC (Fig. 10).

### Relationship between 22 leukocyte subsets and *KLRB1* expression: CIBERSORT method

**Figure 11:**
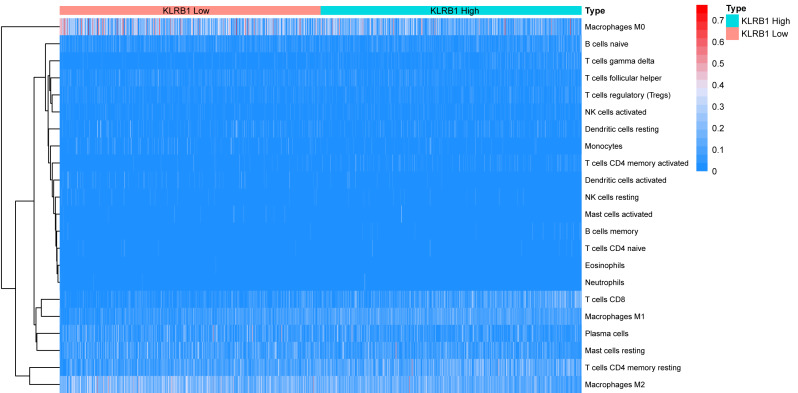
The association between 22 distinct leukocyte subsets and high and low expression groups of KLRB1 shown by heatmap. The high expression group of KLRB1 showed the higher expression of T cells CD8, T cells CD4 memory resting, and macrophages M1, and lower expression of macrophages M0 and macrophages M2.

**Figure 12:**
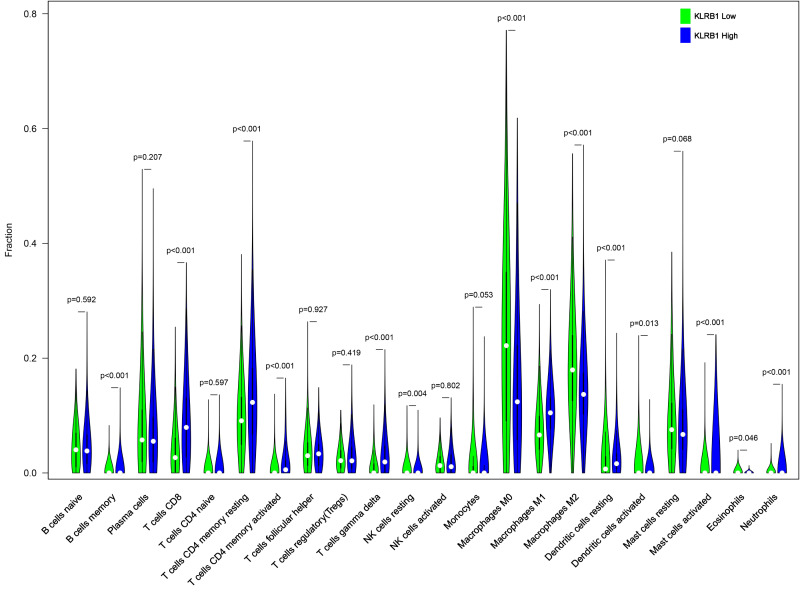
The higher expression could be seen in high KLRB1 group in the subsets of B cells memory (*p* < 0.001), T cells CD8 (*p* < 0.001), T cells CD4 memory resting (*p* < 0.001), T cells CD4 memory activated (*p* < 0.001), T cells gamma delta (*p* < 0.001), macrophages M1 (*p* < 0.001), mast cells activated (*p* < 0.001) and neutrophils (*p* < 0.001), and lower expression in macrophages M0 (*p* < 0.001) and macrophages M2 (*p* < 0.001). .

**Figure 13:**
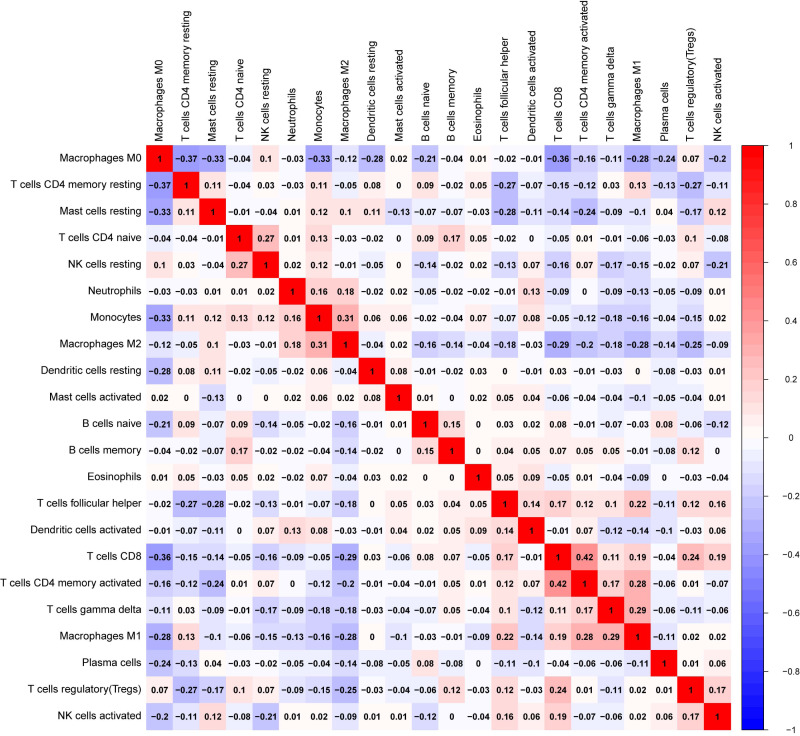
The correlation matrix between each leukocyte subset shown by correlation heatmap in breast cancer. The colored squares are used to display the correlation coefficients (red, positive Spearman’s rho; blue, negative Spearman’s rho).

**Figure 14:**
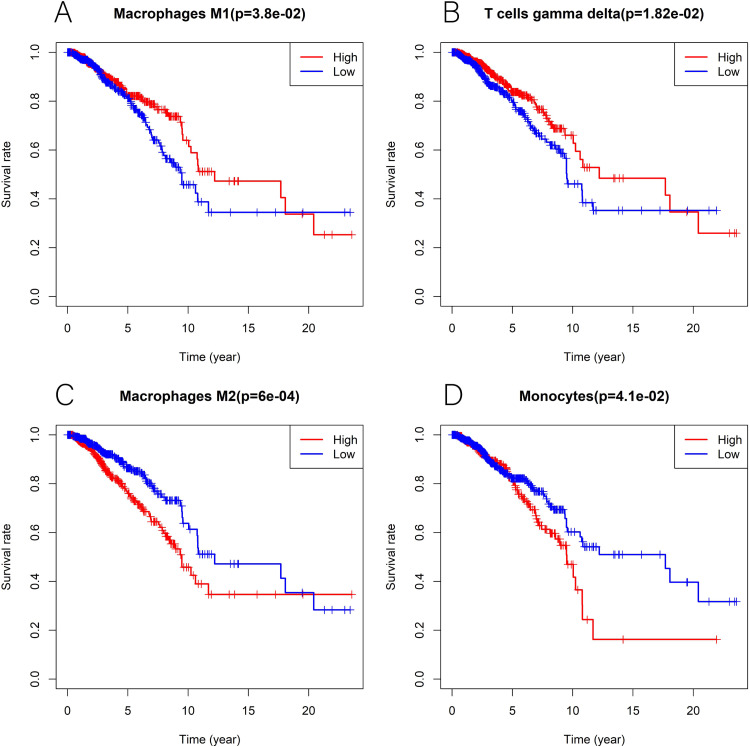
Kaplan-Meier curves in breast cancer according to the high and low expression of distinct leukocyte subsets. The survival analysis showed significantly higher expression of macrophages M1 (A) as well as T cells gamma delta (B), and lower expression of macrophages M2 (C) and monocytes (D) was associated with higher survival rate in breast cancer.

We explored the associations between 22 distinct leukocyte subsets and the high/low *KLRB1* expression groups using the CIBERSORT method. In the high *KLRB1* expression group, M1 macrophages, CD8 cells, and resting CD4 memory cells were highly expressed, while M0 and M2 macrophages were not (Fig. 11). The results were also shown as violin plots. Similarly, a high expression was detected in memory B cells, resting CD4 memory cells, CD8 cells, activated CD4 memory cells, M1 macrophages, gamma delta T cells, activated mast cells, and neutrophils (all *p* < 0.001) *versus* a low expression of M0 and M2 macrophages (both *p* < 0.001) (Fig. 12). The heatmap showed the correlation between each of these 22 leukocyte subsets (Fig. 13). Survival analysis showed that the higher expression of M1 macrophages (*p* = 3.80e−02) and gamma delta T cells (*p* = 1.82e−02) and the lower expression of M2 macrophages (*p* = 6.00e−04) and monocytes (*p* = 4.10e−02) were associated with higher survival rate in BC (Fig. 14).

### GSEA

**Figure 15:**
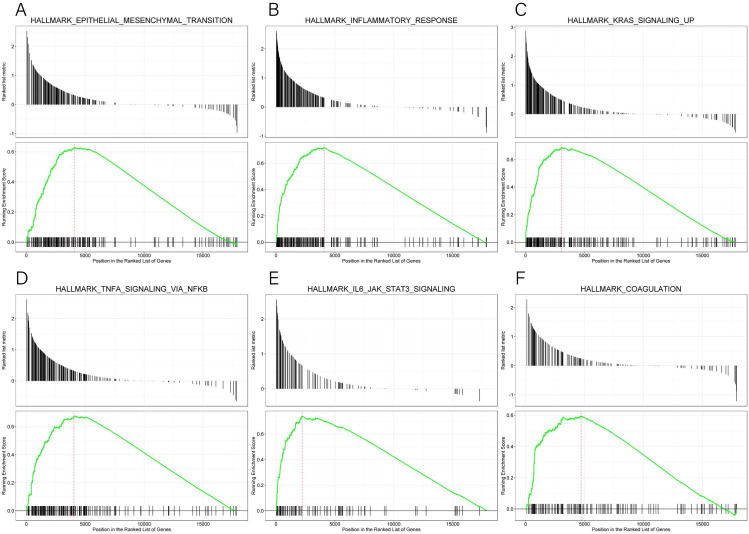
(A–F) Enrichment plots from GSEA. The GSEA results indicated that epithelial mesenchymal transition, inflammatory response, kras signaling up, tnfa signaling *via* NFKB, IL6 JAK STAT3 signaling, and coagulation pathways were differentially enriched in high and low KLRB1 expression groups.

GSEA data and the MsigDB collection were used to identify the enrichment of signaling pathways linked to *KLRB1* expression (NOM *p*-value less than 0.05, FDR less than 0.25). Six pathways (epithelial-mesenchymal transition (EMT), inflammatory response, KRAS signaling up, TNF-*α* signaling *via* NF-*κ*B, IL6-JAK-STAT3 signaling, and coagulation (all *p* < 0.01)) (Table 4, Fig. 15) were differentially enriched in the groups with high/low *KLRB1* expression.

**Table 4:** Gene sets enriched in high phenotype.

Description	NES	*p*-value	*q*-value
HALLMARK_EPITHELIAL_MESENCHYMAL_TRANSITION	1.374	0.001	0.003
HALLMARK_INFLAMMATORY_RESPONSE	1.572	0.001	0.003
HALLMARK_KRAS_SIGNALING_UP	1.503	0.001	0.003
HALLMARK_TNFA_SIGNALING_VIA_NFKB	1.477	0.001	0.003
HALLMARK_IL6_JAK_STAT3_SIGNALING	1.589	0.001	0.003
HALLMARK_COAGULATION	1.297	0.002	0.006

**Notes.**

Gene sets with NOM *p*-value <0.050 and FDR *q*-value <0.250 were considered as significant.

FDR false discovery rate NES normalized enrichment score NOM nominal

### *KLRB1* expression in the tissue sample of BC patients

**Figure 16:**
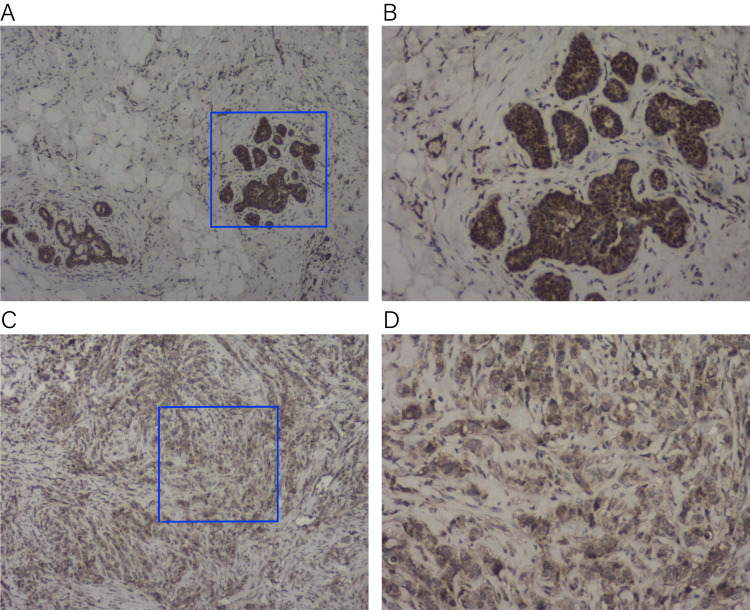
Immunohistochemistry staining for KLRB1. The expression of KLRB1 in breast cancer cell was decreased. (A, B) IHC staining of paratumor breast tissues ((A) × 40, (B) × 100); (C, D) IHC staining of breast cancer tissues ((C) × 40, (D) × 100).

To verify the expression of *KLRB1* among the tissue samples of BC patients (*n* = 23), IHC staining was conducted. *KLRB1* exhibited strong staining in paratumor tissues (Figs. 16A and 16B) and low staining in BC tissues (Figs. 16C and 16D). Cytoplasm and cell membrane showed positive staining for *KLRB1*. Positive staining was found in all paratumor breast tissues, while negative or weak staining was found in 17 tumor tissue, and strong staining was found in six other tissues.

### Correlation between *KLRB1* and genes

Based on the results of correlation analysis, we selected two specific genes to determine their association with BC prognosis. The expressions of forkhead box M1 (*FOXM1)* and transcription factor 12 (*TCF12)* were found to be both significantly and negatively associated with the expression of *KLRB1* (−0.1853 and −0.0825, respectively; Table 5).

**Table 5:** Correlation analysis results of genes.

**Gene 1**	**Gene 2**	**cor**	***p*. value**	***p* adjust**
KLRB1	FOXM1	−0.1853	8.30e−11	2.67e−10
KLRB1	TCF12	−0.0825	4.02e−03	6.37e−03

**Notes.**

Abbreviations: Values of *p* < 0.05 indicate statistically significant correlations.

## Discussion

The association between *KLRB1* expression and the occurrence/development of BC was investigated in this study, particularly *KLRB1*’s impact on TME, relative pathways, and crucial genes. First, pan-cancer analysis was performed. TCGA database analysis showed that in contrast to normal breast tissues, *KLRB1* expression was much lower among BC tissues. Low *KLRB1* levels were linked to poor survival. Univariate and multivariate analyses showed the correlation of *KLRB1* expression with OS and RFS in BC, suggesting that low expression of *KLRB1* could act as a prognosis indicator. These results were validated using GEO datasets and further verified with IHC staining. Moreover, coagulation, EMT, IL6-JAK-STAT3 signaling, inflammatory response, KRAS signaling up, and TNF-*α* signaling *via* NF-*κ*B were enriched in the low *KLRB1* expression group.

The *KLRB1* gene is located on the chromosomal locus 12p13.31 and has a set of several genes of the lectin family of Ca-dependent glycoproteins that are expressed by natural killer cells (Lanier, Chang & Phillips, 1994). It is a type II transmembrane glycoprotein consisting of 225 aa with a molecular weight of 40–44 kDa and a 159-aa extracellular region, which includes a C-type lectin area, a 21-aa transmembrane area, and a 45-aa cytoplasmic portion with a weak immunoreceptor tyrosine-based inhibitory motif (ITIM) (Poggi et al., 1997).

The TME is the environment surrounding a tumor, and its destruction results in cancer progression (Gilmore, 2006). Because of former unsatisfying results regarding the relationship between *KLRB1* and clinical characteristics, we focused on its correlation with the immune mechanism. Single cell analysis results using CancerSCEM showed macrophages to be an important component of TME, having a significant impact on the development of breast tumors. Results of TISCH proved that when compared to stromal cells in BC, *KLRB1* expression in immune cells was relatively higher, implying the vital role of *KLRB1* in the TME of breast tumors. To further explore the relationship between *KLRB1* and the TME, we analyzed immune infiltrating cells (Fig. 8). The expression of *KLRB1* was negatively correlated with the purity of all molecular subtypes of BC. Additionally, a positive association of *KLRB1* expression with the number of infiltrating macrophages, B cells, neutrophils, CD8+ cells, CD4+ cells, and dendritic cells was observed. During monocyte differentiation between bone marrow and precursors in the thymus, *KLRB1* may be expressed on dendritic cells and acquired by cytokines on the surface of NK/T cells (Poggi et al., 1997). Similar to that, the results of this study also proved the positive association between the infiltration of T cells/dendritic cells and *KLRB1* expression.

Furthermore, using the CIBERSORT method, we analyzed the association between leukocyte subset expression and *KLRB1* as well as its impact on the prognosis of BC. The heatmap and violin plots showed the expression of M1 macrophages increased, but M2 macrophage expression decreased in the group with a high *KLRB1* expression compared to the group with low *KLRB1* expression. Based on the correlation heatmap, these two types of macrophages were negatively correlated (−0.28). Tumor-associated macrophages (TAM), which are prominent components of the TME, have been studied extensively and can be categorized into either classically activated (M1) or alternatively activated (M2) macrophages. As pro-inflammatory cells, M1 macrophages contribute to killing cancer cells and have been reported to have antitumor effects, whereas M2 macrophages facilitate tumor growth/metastasis, remodeling of tissues, and immunosuppression, and have been reported to have tumorigenic effects (Biswas & Mantovani, 2010; Gao et al., 2017; Shu et al., 2020). Most malignant tumors are characterized by an increase in glucose uptake and lactate accumulation (Hirschhaeuser, Sattler & Mueller-Klieser, 2011; Meng et al., 2022). Lactate facilitated macrophage differentiation to the M2 phenotype *via* the activation of ERK/STAT3 signaling and promoted angiogenesis, migration, and invasion in BC (Mu et al., 2018). More specifically, G protein-coupled receptor 132 (Gpr132) acted as a crucial sensor of the increased lactate in macrophages and regulated the interaction between BC cells and macrophages during metastasis (Chen et al., 2017). Moreover, M2-like TAM activated BC cells *via* the EGFR/PI3K/AKT signaling pathway and upregulated sodium/glucose cotransporter 1 (SGLT1), leading to tamoxifen resistance and tumor growth (Niu et al., 2021). The NF-*κ*B signaling pathway promotes the differentiation of M1 macrophages in the TME, thereby effectively inhibiting tumor progression (Li et al., 2020). The CIBERSORT analysis results in this study were consistent with the findings above that a higher expression of M1 macrophages and a lower expression of M2 macrophages were noted in the high *KLRB1* expression group *versus* the low *KLRB1* expression group, and these were associated with better OS and RFS. This was consistent with the results shown in Table 2 that among all T stages, T4 was associated with the lowest expression of KLRB1. Similarly, clinical stage IV was associated with the lowest expression of *KLRB1*. Additionally, infiltration by CD8+ and CD4+ led to a favorable prognosis of several tumors (Galon et al., 2006; Zhang et al., 2003).

Immunoreaction analysis showed that *KLRB1* had a positive role in the survival of BC patients. Results of GSEA discovered that low *KLRB1* expression was characterized by the enrichment of TNF-*α* signaling *via* NF-*κ*B, which is an essential protein complex controlling DNA transcription, cytokine production, as well as cell survival (Joyce & Fearon, 2015). NF-*κ*B exerts a pivotal effect on the modulation of immune responses to infection, stimulation, and tumors. However, incorrect regulation, especially sustained activation, can lead to cancer and is regarded as the most common cause of tumorigenesis (Albensi & Mattson, 2000; Meffert et al., 2003). One of the leading mechanisms is the anti-apoptosis effect on tumor cells. Low expression of TNF-*α* (Sheikh & Huang, 2003) could result in the killing of tumor cells, whereas higher levels could lead to cachexia and inflammation (Patel & Patel, 2017). TNF-*α* was negatively associated with *KLRB1*. As the expression of TNF-*α* increased, the NF-*κ*B pathway was overexpressed and constantly activated, leading to the inhibition of TNF-*α* cytotoxicity and apoptosis *via* the constant inhibition of c-Jun N-terminal kinase (Patel & Patel, 2017; Sheikh & Huang, 2003). Therefore, when *KLRB1* expression was low, tumor cell apoptosis was inhibited and the prognosis, as well as survival, was poor. Furthermore, lectin-like transcript-1 (LLT1) was identified as a physiologic ligand for *KLRB1* (identical to NKR-P1A). Functional studies also revealed that the interaction and combination of LLT1 and *KLRB1* could reduce cytokine production in natural killer cells and inhibit TNF-*α* production (Aldemir et al., 2005; Rosen et al., 2005; Rosen et al., 2008), thus verifying the results obtained *via* GSEA. Based on these results, it could be inferred that low expression of *KLRB1*, when acting as an inhibitory receptor, led to a significant production of cytokines and TNF-*α*, possibly owing to the sustained activation of the NF-*κ*B pathway and resulting in poor prognosis.

Data from GSEA confirmed a negative association of *KLRB1* with EMT. The role of EMT in tumor cell progression, invasion, and metastasis has been extensively studied. During transformation, epithelial cancer cells undergo molecular changes and alteration in epithelial features, leading to the acquisition of mesenchymal phenotypes. This transformation promoted the migration as well as invasion of cancer cells. The M1 stage (indicating distant metastasis) was characterized by lower *KLRB1* expression than the M0 stage (Table 2). In addition, EMT was associated with increased enrichment of cancer stem cell-like cells (CSCs), which exhibited mesenchymal characteristics and were resistant to both chemotherapy and targeted therapies (Wu, Sarkissyan & Vadgama, 2016; Yeung & Yang, 2017).

GSEA also showed that IL-6 was upregulated when *KLRB1* was downregulated, and IL-6 played an important role in tumor proliferation, metastasis, and chemotherapy resistance. It was highly expressed in BC, liver cancer, and lung cancer (Mauer, Denson & Brüning, 2015). Low expression of *KLRB1* could lead to high cytokine production (Rosen et al., 2008), and IL-6 was mostly produced by tumor cells as well as tumor-associated fibroblasts. The chronic inflammation in the TME has been reported to facilitate tumor growth as well as induced chemotherapy and radiation therapy resistance (Masjedi et al., 2018). When compared to healthy people, serum IL-6 levels were much higher in ductal carcinomas, indicating that a high level of IL-6 was associated with poor survival (Ma et al., 2017). Furthermore, an advanced study revealed that IL-6 induced EMT in BC cells *via* PIM1, a serine/threonine kinase (Gao et al., 2019). The KRAS signaling up pathway is a BC-associated proto-oncogene, which was also revealed by our GSEA data (Tokumaru et al., 2020).

In a previous study, Gentles et al. and his team (2015) came to the conclusion that *KLRB1* is a significantly favorable prognostic indicator as reported in pan-cancer studies as well as a marker suggesting the characteristics of enhanced innate immunity among diverse T cell subsets (Fergusson et al., 2014). The universally recognized proto-oncogene, *FOXM1*, was not a favorable prognosis indicator (Gentles et al., 2015). More specifically, *FOXM1* is overexpressed in basal-type BC and leads to malignant phenotypes through the upregulation of *AURKB, CCNB1*, and *MYC* and indirect upregulation of *ZEB1* and *ZEB2 via* miR-200b downregulation (Katoh et al., 2013). Combined with the results that *KLRB1* was negatively correlated with *FOXM1*, we demonstrated the linkage between low *KLRB1* expression and poor prognosis in BC patients. Another associated gene, *TCF12*, facilitates cell development and differentiation and probably controls the activation of cancer-associated fibroblasts, which are critical markers of tumor progression. Additionally, TCF12 upregulates fibronectin and lysyl oxidase, triggering BC cell invasion as well as metastasis *in vitro* and *in vivo* (Tang et al., 2016). Therefore, a higher expression of TCF12 contributed to the poor prognosis of patients with BC, which was verified by the negative association of *KLRB1* with TCF12.

This study mainly focused on the association between *KLRB1* expression and BC development and contributed to a better understanding of the importance of *KLRB1* and its potential as a prognosis indicator. Weng et al. (2022) reported similar results that *KLRB1* could serve as a favorable biomarker and may affect tumor immunity. In this study, we begin to explore the differential expression level of *KLRB1* and its effect of overall survival in pan-cancer. Accordingly, our results will further advance *KLRB1* research in BC. One of the unique aspects of our work is that we validated the expression of *KLRB1* at the protein level using IHC in human tissues collected from our center. We additionally found that *KLBR1* can serve as an independent prognosis factor in both OS and RFS, which is more meaningful from a therapeutic perspective. We also conducted an analysis of diagnostic capacity and single cell analysis. Notably, we found that the expression of *KLRB1* was associated with target therapy. The survival analysis determined that the low *KLBR1* group with poorer OS was in the Her-2 positive subtype. Trastuzumab targets Her-2 positive BC cells and may induce antibody-dependent cell-mediated cytotoxicity (ADCC), which involves immune cells such as NK and macrophages. Moreover, we found that M1 macrophages presented more than M2 macrophages in the high *KLRB1* group compared with the low *KLRB1* group, which exhibited significantly better prognosis value. Mu et al. (2018) found that lactate drove macrophage differentiation to the M2 phenotype by the activation of ERK/STAT3 signaling, which promotes angiogenesis, migration, and invasion in breast cancer. These clues hint at the hypothesis that *KLRB1* may be regulated by FOXM1 and TCF12. Moreover, *KLRB1* may affect tumor progression involving RAS/RAF/ERK, STAT3 pathways, immune cells, and glycolysis. However, the above hypothesis requires experimental validation. The previous and our current findings demonstrate a promising direction for future studies.

## Conclusion

This study examined the diagnostic importance of *KLRB1* in patients with BC. Underexpression of *KLRB1* was verified to be an independent biomarker for the prognosis of BC. Additionally, a close relationship between *KLRB1* and the TME, as well as the possibly vital role of *KLRB1* in the TME with a focus on the relevant pathways and critical genes, was also verified. Future experiments (*in vivo* and *in vitro*) may be necessary for the investigation of the biological functions and underlying mechanisms of *KLRB1*.

##  Supplemental Information

10.7717/peerj.15654/supp-1 Table S1 Clinical characteristics of breast cancer patients The numbers of cases in different clinical characteristics categorized by common criterion.

10.7717/peerj.15654/supp-2 File S1 Raw data

10.7717/peerj.15654/supp-3 File S2 Patient information

## Abbreviations

BC: Breast cancer
CLEC5B: C-type lectin domain family 5 member B
CIBERSORT: Cell-type Identification by Estimating Relative Subsets of RNA Transcripts
CTLDs: C-type lectin-like domains
EMT: epithelial mesenchymal transition
ER: estrogen receptor
ERBB2: human epidermal growth factor receptor-2
ESR1: estrogen receptor 1
ESR2: estrogen receptor 2
FDR: false discovery rate
FOXM1: forkhead box M1
GEO: Gene Expression Omnibus
Gpr132: G protein-coupled receptor 132
GSEA: Gene set enrichment analysis
HER-2: human epidermal growth factor receptor 2
HR: hazard ratio
IFN-*γ*: interferon-*γ*
IHC: Immunochemistry
ITIM: immunoreceptor tyrosine-based inhibitory motif
*KLRB1*: killer cell lectin-like receptor B1
LAKs: lymphokine-activated killer cells
LLT1: Lectin-like transcript 1
MHC: major histocompatibility complex
NES: normalized enrichment score
NF-*κ*B: nuclear factor kappa-light-chain-enhancer of activated B cells
NKRP1: natural killer cell surface protein P1
OCIL: osteoclast inhibitory lectin
OS: overall survival
PGR: progesterone receptor
PR: progesterone receptor
RFS: relapse free survival
ROC: receiver operating characteristic
RSEM: RNA-Seq by expectation maximization
SGLT1: sodium/glucose cotransporter 1
TCGA: The Cancer Genome Atlas
TIMER: Tumor Immune Estimation Resource
TNFA: tumor necrosis factor alpha
TCF12: transcription factor 12
TAM: tumor-associated macrophages
TME: tumor microenvironment

## Additional Information and Declarations

### Data Availability

The following information was supplied regarding data availability: The raw data is available in the Supplemental Files.
