# Supplementary material for: The prognostic significance of KLRB1 and its further association with immune cells in breast cancer
Source: PeerJ. 2023 Jul 24;11:e15654. doi: 10.7717/peerj.15654 (PMC10373647; doi:10.7717/peerj.15654)
Supplement: Table S1 — The numbers of cases in different clinical characteristics categorized by common criterion. [file peerj-11-15654-s001.docx]

| Supplementary Table 1 Clinical characteristics of breast cancer patients | |  |
| --- | --- | --- |
| Characteristics | Numbers of cases (%) | |
| Age |  | |
| NA | 2(0.18) | |
| <60  >=60 | 589(53.35)  513(46.47) | |
| Gender |  | |
| NA | 2(0.18) | |
| Female | 1090(98.73) | |
| Male | 12(1.09) | |
| Histological type |  | |
| NA | 3(0.27) | |
| Infiltrating Ductal Carcinoma | 790(71.56) | |
| Infiltrating Lobular Carcinoma | 204(18.48) | |
| Other | 107(9.69) | |
| Molecular subtype |  | |
| NA | 255(23.1) | |
| Basal | 142(12.86) | |
| Her2 | 67(6.07) | |
| LumA | 422(38.22) | |
| LumB | 194(17.57) | |
| Normal | 24(2.17) | |
| ER |  | |
| NA | 50(4.53) | |
| Indeterminate | 2(0.18) | |
| Negative | 239(21.65) | |
| Positive | 813(73.64) | |
| PR |  | |
| NA | 51(4.62) | |
| Indeterminate | 4(0.36) | |
| Negative | 345(31.25) | |
| Positive | 704(63.77) | |
| HER2 |  | |
| NA | 183(16.58) | |
| Equivocal | 180(16.3) | |
| Indeterminate | 12(1.09) | |
| Negative | 565(51.18) | |
| Positive | 164(14.86) | |
| Menopause status |  | |
| NA | 93(8.42) | |
| Inde | 34(3.08) | |
| Peri | 40(3.62) | |
| Post | 706(63.95) | |
| Pre | 231(20.92) | |
| T classification |  | |
| NA | 2(0.18) | |
| T1 | 281(25.45) | |
| T2 | 640(57.97) | |
| T3 | 138(12.5) | |
| T4 | 40(3.62) | |
| TX | 3(0.27) | |
| N classification |  | |
| NA | 2(0.18) | |
| N0 | 516(46.74) | |
| N1 | 367(33.24) | |
| N2 | 120(10.87) | |
| N3 | 79(7.16) | |
| NX | 20(1.81) | |
| M classification |  | |
| NA | 2(0.18) | |
| M0 | 917(83.06) | |
| M1 | 22(1.99) | |
| MX | 163(14.76) | |
| Stage |  | |
| NA | 10(0.91) | |
| I | 182(16.49) | |
| II | 626(56.7) | |
| III | 252(22.83) | |
| IV | 20(1.81) | |
| X | 14(1.27) | |
| Lymph node status |  | |
| NA | 379(34.33) | |
| No | 28(2.54) | |
| Yes | 697(63.13) | |
| Margin status |  | |
| NA | 72(6.52) | |
| Close | 31(2.81) | |
| Negative | 922(83.51) | |
| Positive | 79(7.16) | |
| Vital status |  | |
| NA | 2(0.18) | |
| Deceased | 155(14.04) | |
| Living | 947(85.78) | |
| Radiation therapy |  | |
| NA | 102(9.24) | |
| No | 445(40.31) | |
| Yes | 557(50.45) | |
| Neoadjuvant treatment |  | |
| NA | 3(0.27) | |
| No | 1088(98.55) | |
| Yes | 13(1.18) | |
| Targeted molecular therapy |  | |
| NA | 525(47.55) | |
| No | 46(4.17) | |
| Yes | 533(48.28) | |
| Sample type |  | |
| Metastatic | 7(0.63) | |
| Primary Tumor | 1097(99.37) | |
| OS |  | |
| 0 | 933(85.83) | |
| 1 | 154(14.17) | |
| RFS |  | |
| 0 | 816(89.47) | |
| 1 | 96(10.53) | |
| KLRB1 |  | |
| High | 723(65.49) | |
| Low | 381(34.51) | |

Abbreviation: NA, not available.
